# Novel Possible Protein Targets in Neovascular Age-Related Macular Degeneration: A Pilot Study Experiment

**DOI:** 10.3389/fmed.2021.692272

**Published:** 2022-01-27

**Authors:** Bruno Nobre Lins Coronado, Felipe Bruno Santos da Cunha, Raphaela Menezes de Oliveira, Otávio de Toledo Nóbrega, Carlos André Ornelas Ricart, Wagner Fontes, Marcelo Valle de Sousa, Marcos Pereira de Ávila, Aline Maria Araújo Martins

**Affiliations:** ^1^Department of Medical Science, Faculty of Medicine, University of Brasilia, Brasilia, Brazil; ^2^Faculty of Medicine, CESMAC University Center, Maceio, Brazil; ^3^Department of Health Science, School of Medicine, University Center of Brasilia (UniCEUB), Brasilia, Brazil; ^4^Laboratory of Protein Chemistry and Biochemistry, Department of Cell Biology, Institute of Biological Sciences, University of Brasilia, Brasilia, Brazil; ^5^Faculty of Medicine, Federal University of Goias, Goiania, Brazil

**Keywords:** AMD (age-related macular degeneration), resistance, proteomics, mass spectrometry (MS), biomarkers, choroidal neo vascularization

## Abstract

Age-related macular degeneration (AMD) is among the world's leading causes of blindness. In its neovascular form (nAMD), around 25% of patients present further anatomical and visual deterioration due to persistence of neovascular activity, despite gold-standard treatment protocols using intravitreal anti-VEGF medications. Thus, to comprehend, the molecular pathways that drive choroidal neoangiogenesis, associated with the vascular endothelial growth factor (VEGF), are important steps to elucidate the mechanistic events underneath the disease development. This is a pilot study, a prospective, translational experiment, in a real-life context aiming to evaluate the protein profiles of the aqueous humor of 15 patients divided into three groups: group 1, composed of patients with nAMD, who demonstrated a good response to anti-VEGF intravitreal injections during follow-up (good responsive); group 2, composed of patients with anti-VEGF-resistant nAMD, who demonstrated choroidal neovascularization activity during follow-up (poor/non-responsive); and group 3, composed of control patients without systemic diseases or signs of retinopathy. For proteomic characterization of the groups, mass spectrometry (label-free LC-MS/MS) was used. A total of 2,336 proteins were identified, of which 185 were distinctly regulated and allowed the differentiation of the clinical conditions analyzed. Among those, 39 proteins, including some novel ones, were analyzed as potential disease effectors through their pathophysiological implications in lipid metabolism, oxidative stress, complement system, inflammatory pathways, and angiogenesis. So, this study suggests the participation of other promising biomarkers in neovascular AMD, in addition to the known VEGF.

## Introduction

Age-related macular degeneration (AMD) is expected to affect 288 million people in 2040 (1) and currently represents the primary cause of severe visual loss in patients over 50 years of age in industrialized countries (1–8) with its incidence quadrupling every decade of life (9, 10). Progressive visual impairment in AMD occurs due to degeneration of the photoreceptors and retinal pigment epithelium (RPE) or when new blood vessels form in the center of the retina (6, 11–13). The main symptoms are blurred, distorted, or absent central vision, and patients have problems in reading, driving, and recognizing faces (5, 8, 14).

The most aggressive form of the disease is the neovascular age-related macular degeneration (nAMD), for which an antiangiogenic (anti-VEGF) therapy is considered the gold-standard treatment (15–17). Although anti-VEGF therapy with bevacizumab, ranibizumab, and aflibercept proved important efficacious in the majority of patients, deterioration of visual acuity and persistence of the disease (choroidal neovascularization activity) are observed in about 25% of treated patients (18). These patients represent patients with poor/non-responsive nAMD to anti-VEGF group. Additionally, their definitions are based on morphological and functional criteria or combination of both (18).

Genomics provides important diagnostic information for monogenic diseases and helps to elucidate the risk of developing others. On the other hand, proteomics allows the analysis of the posttranslation profile of numerous diseases, which becomes a strategic tool to assess the severity of the disease and to identify therapeutic targets (19).

The protein global profile of neovascular AMD is not fully defined (20). This study was designed to obtain new information on its pathophysiology, staging (phenotypes), and relative resistance to standard therapy, corroborated with the identification of possible early diagnosis biomarkers and new therapeutic targets, even if as adjuvants.

## Patients and Methods

### Study Design

**Figure 1:**
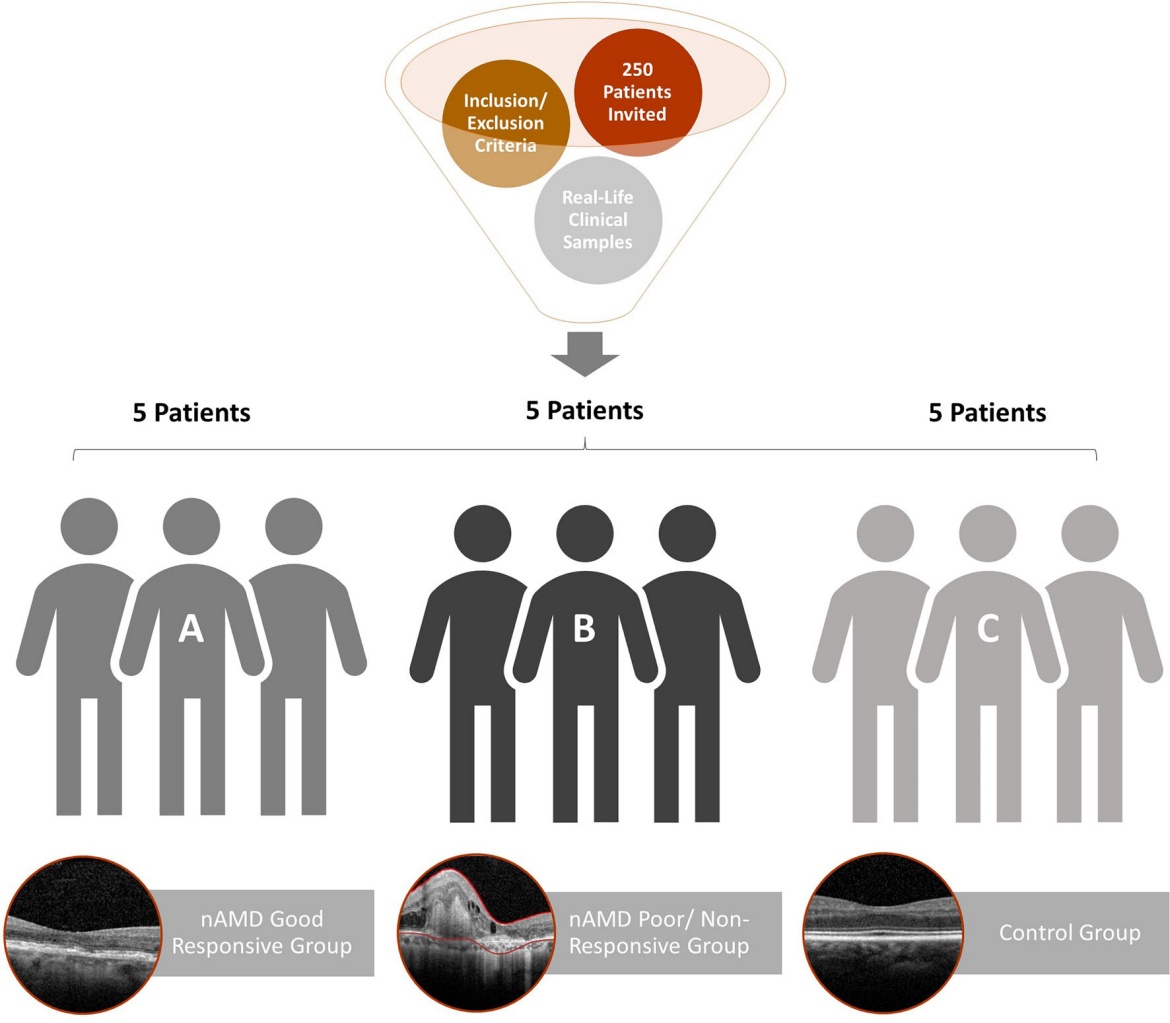
Flowchart of patient recruitment and exclusion for aqueous humor proteome analysis. The 15 patients selected were separated into three groups: **(A)** good response to anti-VEGF therapy (nAMD good responsive); **(B)** resistance to anti-VEGF therapy (nAMD poor/non-responsive); **(C)** control patients without systemic diseases or signs of retinopathy.

**Figure 2:**
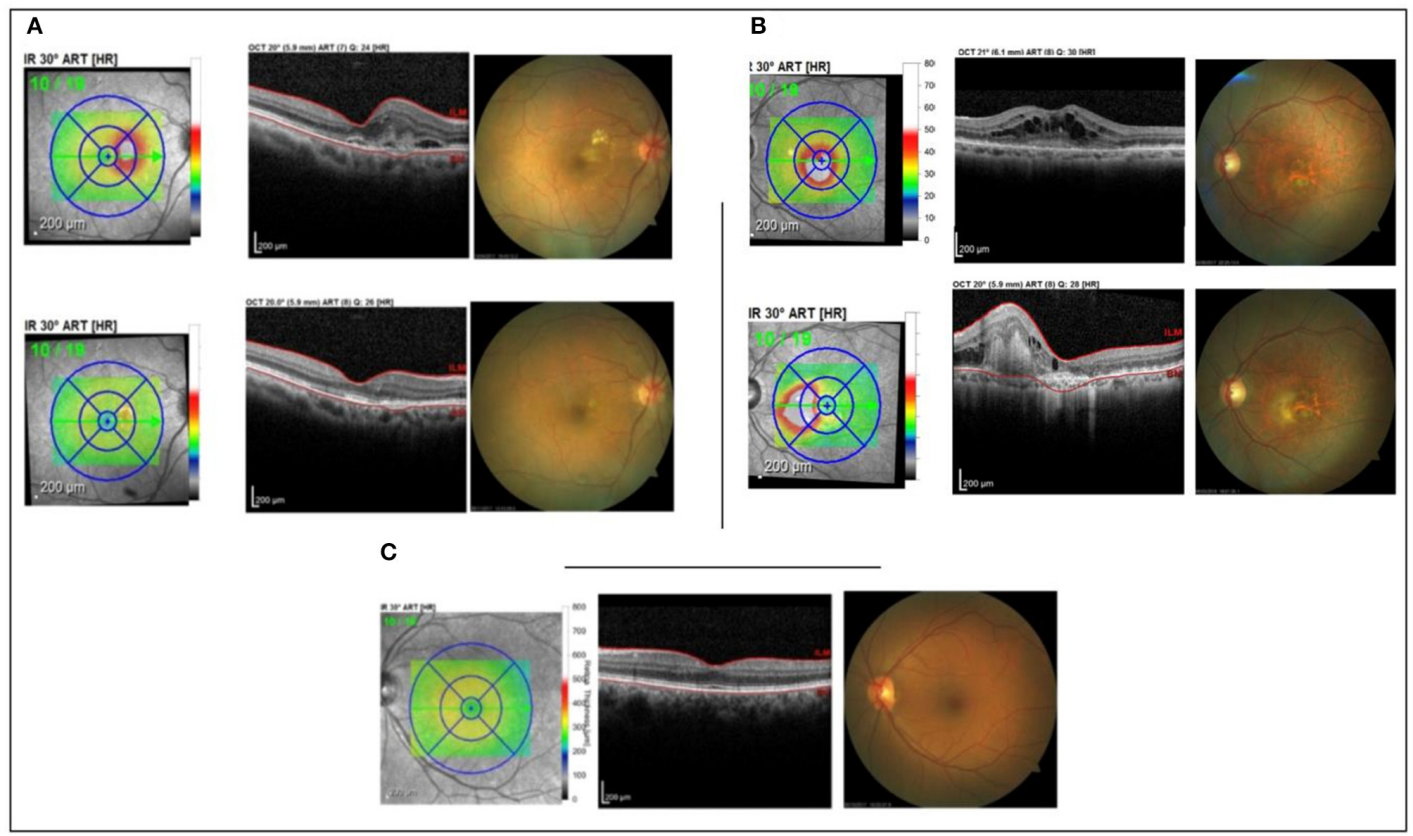
Patient's OCT. **(A)** good responsive group; **(B)** poor/non-responsive group; **(C)** control group.

It is characterized as a pilot study, a prospective and translational experiment in a real-life context. The initial sampling effort, a number of individuals who were invited to participate in the research, was 250 enrolled patients, of which 30 met the inclusion and exclusion criteria (Figure 1). It was previously defined that each group would have five patients with aqueous humor samples collected at the Brazilian Vision Center (Brasília, Brazil), from January 17 to July 18. In the follow-up of these patients, the response to 06-month intravitreal injections of anti-VEGF was analyzed for retinography and spectral-domain optical coherence tomography (OCT) (Figure 2). Due to the response to anti-VEGF therapy, the first 5 that presented a good response to treatment formed group 1 (good responsive), the first 5 that presented poor or non-response formed group 2 (poor/non-responsive). Group 3 (control group) was composed of 5 patients with cataracts, without systemic diseases or signs of retinopathy (Table 1).

**Table 1:** Clinical date of the groups of patients enrolled in the study.

	**Group 1: Good responsive**	Group 2: Poor/Non-responsive^*^	**Group 3: Control**	***P*-value**
Age (yr)	72.8 ± 11.9	81.4 ± 4.3	73.0 ± 6.9	0.24^†^
Sex	Female: 60%(3/5) Male: 40% (2/5)	Female: 80% (4/5) Male: 20% (1/5)	Female: 60% (3/5) Male: 40% (2/5)	0.74^‡^
Race	White: 80% (4/5) Other: 20% (1/5)	White: 60% (3/5) Other: 40% (2/5)	White: 60% (3/5) Other: 40% (2/5)	0.74^‡^
Central Retinal Thickness (CRT), μm	349.8 ± 99.2	334.6 ± 94.2	289 ± 6.7	0.58^†^
Visual-acuity Decimal notation and Snellen equivalent (R: right/L: left)	0.20 ± 0.18	0.30 ± 0.22	0.20 ± 0.19	0.53^†^

* *Response to anti-VEGF injections in nAMD patients*.

† *ANOVA*.

*Chi-square*.

#### Ethics Statement

This research was approved by the Institution's Research Ethics Committee (CAAE 64921317.1.0000.5667–Platafoma Brazil). Written informed consent was obtained from the individuals for the publication of any potentially identifiable images or data included in this article.

#### Inclusion Criteria

These included patients with AMD without other ocular and systemic comorbidities. The patients who were included in this real-life study were classified according to the functional (visual acuity) and morphological (presence/absence of subretinal fluid, intraretinal fluid, intra-retinal cysts, and increase in central retinal thickness) criteria, evaluated by clinical examination and by spectral domain OCT [Amoaku et al. (21), Supplementary Table S1]. Follow-up was at least 6-month injections of anti-VEGF (ranibizumab 0.5 mg/0.05 ml or aflibercept 2 mg/0.05 ml). The best-corrected visual acuity (MAVC) was between 20/25 and 20/400 according to the Snellen table.

#### Exclusion Criteria

These included patients who presented signs of retinopathy other than AMD. Patients with senile cataract (physiological lens aging) within the control group, who showed any signs of evident retinopathy, were excluded. All patients with a history of intraocular surgery other than anti-VEGF injections in the 6 months prior to the start of the research were excluded.

#### Sample Collection

The sample was 0.05 ml of aqueous humor collected by anterior chamber paracentesis, using an ultrafine 30-G disposable syringe needle, before the application of anti-VEGF, performed by an experienced surgeon, in an operating room.

### Sample Preparation

Samples were diluted in 0.02M TEAB solution and sonicated with 40% intensity in 3 cycles of 10 s each using a Tip Sonicador Q125 (QSonica). Protein concentrates were quantified using Qubit™ method (Invitrogen). Aliquots containing 10 μg of protein were submitted to trypsin in-solution digestion, and the resulting peptide samples were desalted using homemade C18 reverse-phase microcolumns (22). The peptide concentration was also determined using Qubit™ protein assay.

### Mass Spectrometry

The LC-MS/MS analyses were performed on a Dionex Ultimate 3000 RSLCnano UPLC system (Thermo Fisher Scientific) coupled to an LTQ-Orbitrap Elite mass spectrometer (Thermo Fisher Scientific). Aliquots containing 1 μg of peptides were injected into a homemade precolumn (100 μm internal diameter x 3 cm long) with spherical silica particles coated with 5 μm C18 Reprosil-Pur and 120Å pores to remove salt residues. Subsequently, these peptides were fractionated in a homemade analytical column (75 μm internal diameter x 24 cm long) with 3 μm C18 Reprosil-Pur particles and 120Å pores. The linear elution gradient between solvents A (0.1% formic acid in water) and B (0.1% formic acid in acetonitrile) was composed of 2 to 35% B over 155 min. Mass spectra were acquired in positive mode with data-dependent MS/MS spectra acquisition (DDA). In MS1, high-resolution spectra (120000 FWHM) between 300 and 1,650 m/z were obtained in the Orbitrap analyzer. Each scan was followed by MS2 in the ion trap analyzer of the 20 most intense ions above the required minimum signal of 3,000 by the collision-induced dissociation (CID) method. The reanalysis of already fragmented ions was inhibited by dynamic exclusion, thus favoring the identification of less abundant peptides.

### Data Analysis

Spectra were processed by Progenesis QI software (http://www.nonlinear.com/progenesis/qi) (Nonlinear Dynamics ©), where they were first submitted to the chromatographic retention time alignment and then quantified according to the area of integrated intensity of the peaks recovered from the extracted ion chromatogram (XIC). Statistically significant events (ANOVA *p* ≤ 0.05) had their MS2 spectra exported for the identification on the Peaks® Studio 7.0 platform (http://www.bioinfor.com/peaks-studio) (Bioinformatics Solutions, Inc.) obeying the following parameters: proteins with a minimum of 2 peptides, homo sapiens taxonomy, UniProtKB database–SWISS-PROT/TrEMBL (April/2019), MS1 accuracy of 10 ppm; MS2 accuracy of 0.5 Da; up to two missed cleavage sites; carbamidomethylation of cysteines as fixed modification; methionine oxidation as a variable modification. Number and groups of proteins and number of peptides were filtered with false-discovery rate (FDR) < 0.01 before the identification list was exported to Progenesis QI for analysis of variance (*p* ≤ 0.05), PCA and dendogram. MetaboAnalyst 4.0® (http://www.metaboanalyst.ca) (23) and String (https://string-db.org/) (24) platforms completed the data refinement based on the experimental design. To unify gene representations and the attributes of the various gene products, the proteins identified in this study were submitted to gene ontology (GO term) for molecular function, biological process, and analysis of cellular components.

## Results

**Figure 3:**
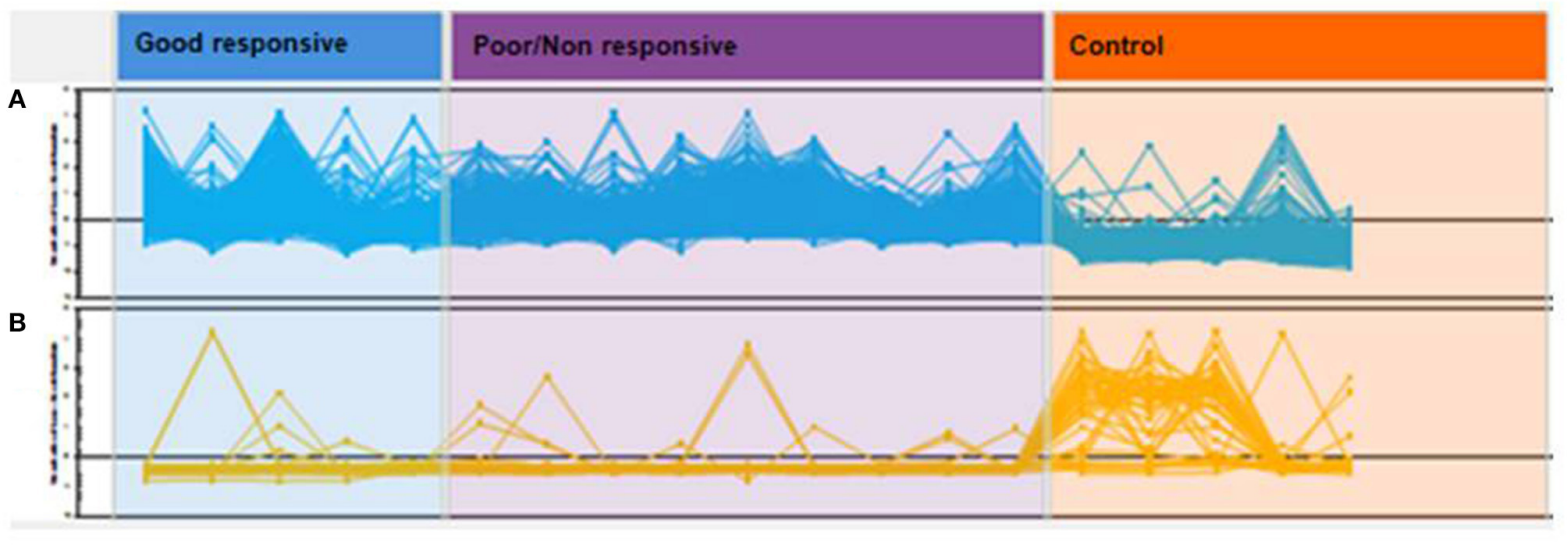
Hierarchic cluster analyses (HCA) of global protein profiles. Each line represents a protein and each peak or valley, its abundance per patient. Proteins in **(A)** have a predominance of positive regulation in cases of good responsive nAMD and poor/non-responsive nAMD with the predominance of negative regulation in controls. In **(B)**, the opposite relationship is observed.

**Figure 4:**
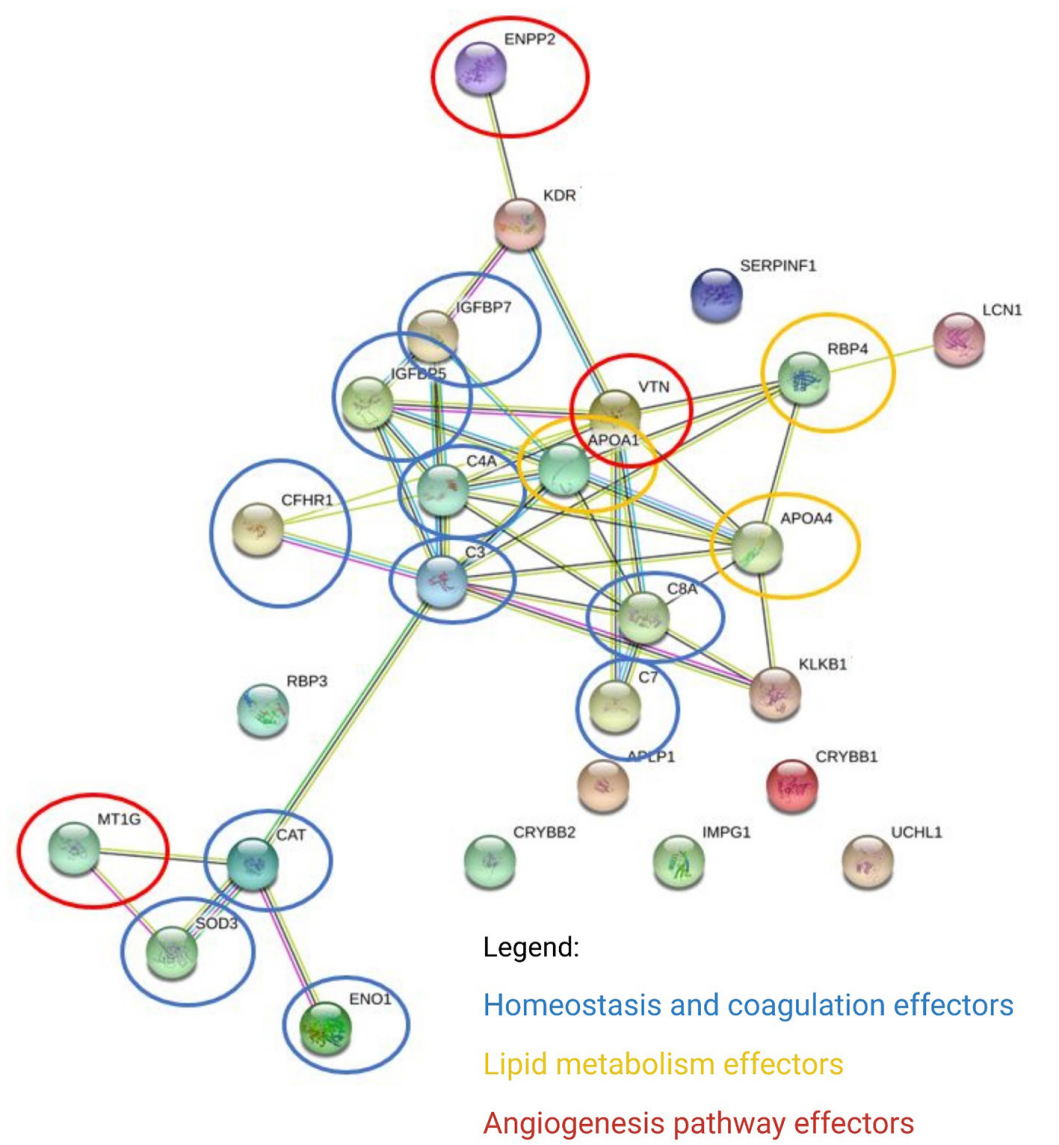
Differentially expressed proteins converge on main pathways by biological processes. The links between proteins represent interactions, according to the pattern of the String program (protein-protein interaction network enriched with functional analysis). ENPP2, ectonucleotide pyrophosphatase/ phosphodiesterase family member 2; KDR, kinase insert domain receptor (A type III receptor tyrosine kinase), isoform CRA_a; IGFBP7, insulin-like growth factor-binding protein 7; IGFBP5, insulin-like growth factor-binding protein 5; CFHR1, complement factor H-related protein 1; C4A, complement C4-A; APOA1, apolipoprotein A-I, isoform CRA_a; VTNC, vitronectin; C3, tetranectin; C8A, complement component C8 alpha-chain; APOA4, apolipoprotein A-IV; RBP4, retinol-binding protein 4; SERPINF1, pigment epithelium-derived factor; LCN1, lipocalin 1 (tear prealbumin), isoform CRA_a; C7, complement component C7; APCP1, amyloid-like protein 1; CRYBB2, beta-crystallin B2; IMPG1, interphotoreceptor matrix proteoglycan 1; CRYBB1, beta-crystallin B1; UCHL1, ubiquitin carboxy-terminal hydrolase; KLKB1, plasma kallikrein; RBP3, retinol-binding protein 3; CAT, cathepsin D; MT1G, metallothionein 1G; SOD3, extracellular superoxide dismutase [Cu-Zn]; ENO1, enolase 1 (alpha), isoform CRA_a.

**Figure 5:**
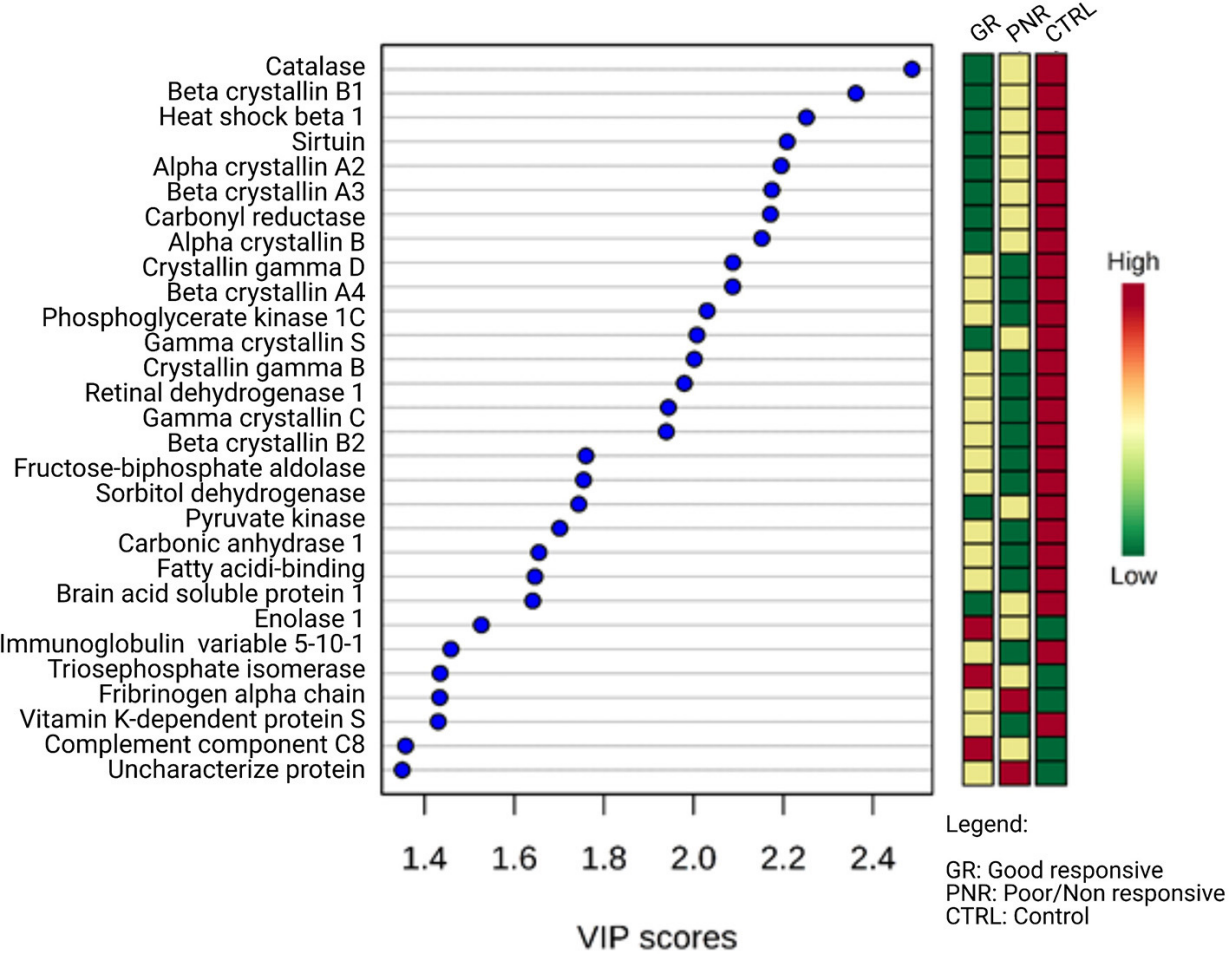
Multivariate analyses by VIP scores of regulated proteins in aqueous humor of patients with good responsive nAMD, poor/non-responsive nAMD, and control patients (PLS-DA imp. features). Among the 30 regulated proteins with the highest VIP score, 25 have negative regulation in pathological scenarios. The colored boxes on the right indicate the relative intensity of each protein in the respective scenarios.

In the aqueous humor samples analyzed, a total of 2,336 proteins were identified, using an FDR 1% of which 185 were statistically significant (ANOVA *p* < 0.05) and allowed the distinction between the three groups analyzed in this study (Figure 3). Thirty-nine proteins were correlated with visual function or with metabolic pathways directly or indirectly linked to choroidal neoangiogenesis, an essential condition for neovascular AMD (Table 2). Additionally, according to the String program pattern, most of the selected proteins play important roles at a functional protein-protein interaction network (Figure 4). Using supervised multivariate analysis, as partial least squares discriminant analysis (PLS-DA), it was possible to obtain the variable importance projection scores (VIP scores) and identify the 30 proteins which are most discriminants within each scenario (Figure 5).

**Table 2:** Selected discriminant proteins by metabolic function related to the pathophysiology of AMD.

**Protein**	**Important Functions/Biological Process**	**Regulated Positively**	**Regulated Negatively**	**Anova (p)**
**VISUAL FUNCTION**				
Alpha-crystallin B chain (CRYAB)	- structural constituent of eye lens			
tblbreak - visual perception - amyloid beta binder - response to hypoxia	Control	*Good Responsive*	0.0004	
Beta-crystallin A4 (CRBA4)	- visual perception - structural constituent of eye lens	Control	*Good Responsive*	0.00001
Beta-crystallin B1 (CRBB1)	- visual perception - structural constituent of eye lens	Control	*Good Responsive*	0.0000002
Beta-crystallin B2 (R4UMM2)	- visual perception - structural constituent of eye lens	Control	*Good Responsive*	0.0001
Crystallin, beta B3, isoform CRA_a (CRYBB3)	- visual perception - structural constituent of eye lens	Control	*Good Responsive*	0.01
Gamma-crystallin C (CRYGC)	- visual perception - structural constituent of eye lens	Control	Poor/Non-Responsive	0.0003
Interphotoreceptor matrix proteoglycan 1 (IMPG1)	- visual perception	*Good Responsive*	Control	0.0002
Retinol-binding protein 4 (RET4)	- visual perception - retinal binding - retinol binding	*Good Responsive*	Control	0.01
**LIPID METABOLISM**				
Apolipoprotein A-I, isoformCRA_a (APOA1)	- amyloid-beta binding - cholesterol transfer activity - high-density lipoprotein particle binding - identical protein binding - lipase inhibitor activity - lipoprotein biosynthetic process	Poor/Non-Responsive	Control	0.0004
Apolipoprotein A-IV (APOA4)	- antioxidant activity - cholesterol binding - copper ion binding - lipid binding - lipid homeostasis	Poor/Non-Responsive	Control	0.0001
Phosphoinositide phospholipase C (Q86YU9)	- lipid catabolic process	Poor/Non-Responsive	Control	0.01
Retinol-binding protein 3 (RBP3)	- retinal binding - retinol binding - retinoid metabolic process - visual perception - lipid metabolic process	Poor/Non-Responsive	Control	0.002
**OXIDATIVE STRESS**				
Amyloid-likeprotein 1 (APLP1)	- heparin binding - transition metal ion bond	*Good Responsive*	*Control*	0.02
Catalase (CAT)	- cellular response to oxidative stress - negative regulation of the apoptotic process	*Control*	*Good Responsive*	0.0004
Enolase 1 (HEL-S-17)	- autoimmune stimulation	Control	Poor/Non-Responsive	0.003
Glutathione peroxidase (GPX3)	- glutathione peroxidase activity - response to oxidative stress	Poor/Non-Responsive	*Control*	0.03
Lipocalin 1 (Tear prealbumin), isoformCRA_a (CRA_a LCNI)	- small molecule binding - modulation of oxidative stress	*Good Responsive*	Poor/Non-Responsive	0.02
Extracellular superoxide dismutase [Cu-Zn] (SOD)	- cellular response to oxidative stress - response to hypoxia	Poor/Non-Responsive	*Control*	0.001
**COMPLEMENT SYSTEM ACTIVATION**				
Immunoglobulin heavy constant mu (IGHM)	- adaptive immune response - B cell receptor signalingpathway - classic complement pathway activation - innate immune response - leukocyte migration - positive regulation of B cellactivation	*Good Responsive*	Poor/Non-Responsive	0.01
CFB (A0A1U9X7H5)	- complement activation	Control	Poor/Non-Responsive	0.04
Clusterin (A0A384NKS6)	- inflammatory response - complement system regulation	Poor/Non-Responsive	Control	0.0009
Complement C2 (B4DQI1)	- complement activation	Poor/Non-Responsive	Control	0.03
Complement C3 (CO3)	- complement activation	Poor/Non-Responsive	Control	0.0002
Complement C4-A (CO4A)	- complement activation - positive regulation of apoptotic cell clearance	Poor/Non-Responsive	Control	0.0002
Complement C7 (CO7)	- complement activation	Poor/Non-Responsive	Control	0.0004
Complement component C8 alpha chain (CO8A)	- complement activation	*Good Responsive*	Control	0.01
Complement factor H-related protein 1 (CFHR1)	- complement activation - regulation of complement activation	*Good Responsive*	Control	0.004
Vitronectin (VTNC)	- regulation of complement activation - positive regulation of VEGF	Poor/Non-Responsive	Control	0.00001
**INFLAMMATION**				
Monocyte differentiation antigen CD14 (F1C4A7)	- inflammatory response - innate immune response	Poor/Non-Responsive	Control	0.0003
Immunoglobulin heavy constant mu (IGHM)	- adaptive immune response - B cell receptor signaling pathway - classic complement pathway activation - innate immune response - leukocyte migration - positive regulation of B cell activation	*Good Responsive*	Poor/Non-Responsive	0.01
Plasma kallikrein (KLKB1)	- serine-type endopeptidase activity - inflammatory regulation - disassembly of the extracellular matrix - fibrinolysis - plasminogen activation	Poor/Non-Responsive	*Good Responsive*	0.03
Clusterin (A0A384NKS6)	- inflammatory response - complement system regulation	Poor/Non-Responsive	Control	0.0009
Enolase 1 (HEL-S-17)	- autoimmune stimulation	Control	Poor/Non-Responsive	0.003
Pigment epithelium-derived factor (PEDF)	- negative regulation of angiogenesis - negative regulation of the inflammatory response	*Good Responsive*	*Control*	0.03
**ANGIOGENESIS**				
Ectonucleotide pyrophosphatase /phosphodiesterase family (ENPP2)	- regulation of angiogenesis	*Good Responsive*	*Control*	0.003
Insulin-like growth factor-bindingprotein 7 (IGFBP7)	- regulation of angiogenesis - immune response	*Good Responsive*	*Control*	0.0006
Insulin-like growth factor binding protein 4, isoformCRA_a (IGFBP4)	- insulin-like growth factor binding - inflammatory response - positive regulation of insulin-like growth factor receptor signaling pathway	*Good Responsive*	*Control*	0.0007
Pigment epithelium-derived factor (PEDF)	- negative regulation of angiogenesis - negative regulation of the inflammatory response	*Good Responsive*	*Control*	0.03
Tissue inhibitor of metalloproteinase 1 (TIMP 1)	- regulation of angiogenesis -metalloendopeptidase inhibitor activity	Poor/Non-Responsive	*Control*	0.002
Metallothionein-1G (MT1G)	- cellular response to stimulation of vascular endothelial growth factor	*Control*	*Good Responsive*	0.001
Vascular endothelial growth factor receptor 1 (FLT1, VEGFR-1)	- ATP binding - vascular endothelial growth factor-activated receptor activity - regulation of angiogenesis	Poor/Non-Responsive	*Good Responsive*	0.004
Kinase insert domain receptor (A type III receptortyrosinekinase), isoform CRA_a (KDR)	- regulation of angiogenesis	Poor/Non-Responsive	*Good Responsive*	0.03
Vitronectin (VTNC)	- regulation of complement activation - positive regulation of VEGF	Poor/Non-Responsive	Control	0.00001
Ubiquitin carboxyl-terminal hydrolase (V9HW74)	- response to ischemia	Control	*Good Responsive*	0.01

*Observation: Response to anti-VEGF injections in nAMD patients. Thirty-nine proteins correlated with visual function or with meta-bolic pathways directly/indirectly linked to choroidal neoangiogenesis. The redundancy of some proteins is due to the characteristic in which some of these effectors display in multiple cellular functions*.

A posteriori statistical power analysis demonstrated that five patients per group were sufficient to achieve statistical robustness, with all samples having a statistical power > 0.8 (86.4%), which means that the experimental design allowed 84% of the data to present statistical power >0.8.

## Discussion

In addition to the vascular endothelial growth factor (VEGF) pathway, some other pathophysiogenic pathways in nAMD are described in the literature, and choroidal neoangiogenesis has been already correlated with oxidative stress, complement system activation, immune system activation, and lipid metabolism (25–28). This study was able to identify 39 proteins related to visual function or to at least one alternative pathophysiological pathway of neovascular AMD (Table 2).

Drusen, one of the main signs of AMD, correspond to an accumulation of cellular debris, including proteins, minerals, and lipids, between Bruch's membrane and the RPE. Thompson et al. (29) proposed a new mechanism for drusen formation and growth. They confirmed the presence of calcium phosphate (hydroxyapatite, HAp) in small hollow spheres containing cholesterol in all sub-RPE deposits in the macula and in the periphery. Using immunohistochemistry, they showed that numerous proteins coated those small spheres, such as complement factor H, vitronectin (VTNC), and beta-amyloid. Lipids and lipoproteins that contain cholesterol, which is produced and secreted at least in part by the RPE (30), are recruited and retained in the aged Bruch's membrane (31, 32), which in turn, in a self-directed oligomerization process, facilitates additional depositions that ultimately lead to drusen formation and growth (33). Studies have also established analogies between the oxidation of lipoproteins in Bruch's membrane and atherosclerosis (33–37).

Drusen function as a barrier between the choriocapillary layer and the photoreceptors, limiting the oxygenation and nutritional support of cones and rods and generating local hypoxia, a fundamental agent in the pathophysiogenesis of AMD (38).

### Physiopathological Pathways Described for AMD

#### Visual Function

Crystallins, the main components of the lens, also act in the regulation of astrocytes, the remodeling of retinal vessels (39), the regulation of apoptosis (40), and the depuration of the external segments of photoreceptors by the RPE (41). In neovascular AMD, some serum markers (42) and numerous autoantibodies and immune complexes against α- and β-crystallins have been demonstrated in the retina and choroid (43). The α-crystallins are expressed in the cytosol and mitochondria of RPE cells, protecting them from oxidative stress. They function as angiogenic and VEGF modulators. Chaperone proteins derived from α-crystallins function as inhibitors of oxidation-induced cell death (44). The β-crystallin proteins and interphotoreceptor matrix proteoglycan 1 (IMPG1), reported in this study and involved in visual perception, allowed the grouping of biological replicates and the distinction between the three research groups: good responsive to anti-VEGF, poor/non-responsive to anti-VEGF, and control. The literature lacks studies that establish a consistent correlation of these proteins with the pathophysiogenesis of AMD. Retinol-binding protein 4 (RBP-4) not only has GO term annotations for its involvement in visual perception and the metabolism of retinol, but it has been described as a potential target in treating atrophic AMD and Stargardt's disease (45).

#### Lipid Metabolism

There are in the literature reports which demonstrate that lipid accumulation in the formation of drusen can be considered as an early event in the pathophysiogenesis of AMD (46). Thus, in senile patients, oxidized lipoproteins and lipids can be considered as triggers of AMD (8, 33, 34).

This study reported numerous proteins frequently associated with lipoprotein particles, including complement factor H (CFHR1), amyloid-similar protein 1 (APLP1), and VTNC, which were highlighted by Thompson et al. (29) as having roles in the pathophysiogenesis of AMD. Moreover, it was possible to demonstrate that the A-I and A-IV apolipoprotein isoforms were upregulated in patients with poor/non-responsive AMD, thus corroborating the results of Levy et al. (47) who proved that apolipoprotein is a factor that promotes the survival of subretinal mononuclear phagocytes and therefore stimulates chronic inflammation in AMD. Phosphoinositide phospholipase C (Q86YU9) and retinol-binding protein 3 (RBP-3), which are related to lipid metabolism, were reported in the analyzed samples, and they were upregulated in the poor/non-responsive group and downregulated in the control group. Morohoshi et al. (48) identified antiretinal antibodies against RBP-3 in patients with neovascular AMD. Recent studies in the literature demonstrate the potential strategic importance of other antibodies, such as anti-HtrA1, in the therapy of AMD geographic atrophy (49).

#### Oxidative Stress

The association between oxidative stress and age-related conditions has been extensively documented, as in Alzheimer's disease, Parkinson's disease, atherosclerosis, certain types of cancer, and AMD (50–52). For comparison, the retina has the highest oxygen consumption per gram of tissue in the human body, and along with the RPE, it is extremely susceptible to damage because of oxidative stress (53). When the antioxidant capacity of the eye is exceeded, a large number of oxidized proteins, lipids, and inflammation-related factors are formed, which cause drusen to appear (54). The heterophagy of photoreceptors by RPE cells is a constant source of polyunsaturated fatty acids, thus generating an environment rich in reactive oxygen species (ROS) (55) that can induce the oxidative modification of phospholipids. ROS exposure induces immune recognition with inflammatory damage, primarily through complement activation (53, 56).

This study identified several proteins related to redox imbalance as amyloid protein (APLP1), catalase (CAT), enolase (HEL-S-17), glutathione peroxidase (GPx), lipocalins (CRAa LCNI), and superoxide dismutase (SOD) regulated within the biological scenarios, which allowed distinct protein clusters in all three phenotypes. A correlation was observed between these proteins' GO annotations and functional changes caused by oxidative stress and present in pathophysiology of AMD.

The retina contains a considerable number of antioxidant agents in photoreceptors and the RPE, primarily in the central portion of the retina, including SOD, GPx, and CAT (57). Increased concentrations of SOD and GPx were reported in the anti-VEGF poor/non-responsive group and CAT was upregulated in the control group. That is, catalase, as one of the most important antioxidant enzymes, was reduced in patients with AMD in this research, a condition compatible with the literature whose CAT deficiency or malfunction is related to many degenerative diseases associated with age, such as diabetes mellitus, vitiligo, cardiovascular diseases, Wilson's disease, hypertension, anemia, some dermatological disorders, Alzheimer's disease, bipolar disorder, and schizophrenia (58). SOD and GPx are produced in an oxidative injured environment, whereas CAT is a physiological constituent of the cell.

Amyloid protein, known to be present in Alzheimer's disease (59), is another important component of drusen (60, 61), and in this study, it was reported to be downregulated in the control group (patients without AMD). Lipocalin 1 (LCN1), which has already been characterized as a protector against the oxidative stress, potentially because of lipid peroxidation products, was reported in this study to be upregulated in patients with good responsive AMD and downregulated in patients with anti-VEGF poor/non-responsive.

Enolase (HEL-S-17) is a key enzyme of the glycolytic pathway that is reported in AMD geographic and neovascular atrophies (48, 62, 63), and has GO annotations correlating it with oxidative stress. Enolase was reported to be upregulated in the control group and downregulated in the anti-VEGF poor/non-responsive group. Several researchers have described autoimmune stimulation of enolase, and its serum measurement is a potential staging or, even, a prognostic tool for AMD. Adamus et al. (62) have analyzed antiretinal autoantibodies against enolase at different stages of AMD.

#### Complement System

Over the years, photoreceptors and RPE cells are exposed to numerous innate immune activators. Therefore, a strict regulation of immunity is essential to prevent harmful inflammatory events. However, the deregulation of the complement system can potentially lead to chronic inflammation of the eye. One of the first indications that the complement system is involved in the progression of AMD was the identification of complement by-products (factor H) in drusen (64). In this study, an increase in C2, C3, C4, and C7 was reported in patients with poor/non-responsive AMD, in accordance with the literature (25, 65, 66). Factor C8 was reported to be upregulated in the group of good responsive AMD. As reported by Lu et al. (66), complement factor B (CFB) was reported in a higher concentration in control patients, functioning as a preventive factor for AMD.

Clusterin (CLU), according to the observed GO annotations, is involved in inflammatory processes and in the complement cascade. However, it can play a protective role in the response to the redox (oxidative stress) environment of human RPE cells, contributing to cell survival using PI3K/Akt, an intracellular signaling pathway with an important role in the regulation of the cell cycle. Therefore, CLU can be considered as a preventive factor for AMD. It was annotated in a relatively greater concentration in patients with poor/non-responsive AMD in this study and which could demonstrate that this pathway remains active in an attempt to restore local homeostasis. Similar to APLP1, CLU has been described in the pathophysiogenesis of Alzheimer's disease (67). The heavy chain of immunoglobulin Mu (IGHM), in turn, despite GO annotations correlating it with the immune response and complement activation, was a marker found in this study, although no association with AMD has been described in the literature until nowadays.

However, VTNC, which has been associated with lipoprotein particles, has GO annotations within extracellular matrix (ECM) constituents, upregulation of VEGF (angiogenesis), cell adhesion, and complement regulation. It is also considered one of the main proteins involved in the formation of drusen (68).

Numerous studies confirm the close relationship of the complement system with the progression of retinal degeneration (69). However, the adaptive immune response that leads to the activation and regulation of inflammatory cytokines of B and T cells has not yet been fully elucidated (70, 71).

#### Inflammation

The heavy chain of IGHM, CLU, and enolase (HEL-S-17) are some of the proteins marked in this study that are potentially involved in inflammatory pathways and are linked to other pathways of neovascular AMD. The CD14 monocyte differentiation antigen (F1C4A7) and plasma kallikrein (KLKB1) were reported to have different regulation levels throughout the groups analyzed in this study. Most significantly, they were upregulated in the poor/non-responsive AMD group. However, the literature lacks studies that show a direct correlation of these proteins with AMD.

Guo et al. (72) and Ali et al. (73) inferred a correlation between CD14 antigen with apolipoprotein E (APOE), the main lipoprotein found in the retina (72–74), which plays a crucial role in local lipid transport (47). Thus, the CD14 antigen can also potentially participate in lipid metabolism in the pathogenesis of AMD.

#### Angiogenesis

The impairment of RPE function is an early and crucial event that influences and drives choroidal neovascularization (75, 76). The pigment epithelium-derived factor (PEDF) participates in the suppression of angiogenesis and negatively regulates the inflammatory response. It was found to be negatively regulated in the control group (cataract) in this study, precisely in an environment with reduced need for inflammatory and angiogenesis inhibiting factors.

Insulin-like growth factor-binding protein 7 (IGFBP-7) and PEDF are inhibitors of angiogenesis and were upregulated in patients with neovascular AMD. Ectonucleotide pyrophosphatase (ENPP2) was upregulated in the good responsive group, but despite having a GO annotation correlating it with the neoangiogenesis, scarce literature correlates this protein with AMD.

The growth of choroidal neovascularization through the Bruch's membrane is stimulated by producing metalloproteinases (MMPs), which are enzymes that degrade ECM proteins (75). Tissue inhibitors of metalloproteinases (TIMPs) also play an important role in the degradation of ECM components or in the accumulation of deposits in the Bruch's membrane (BM) (77). By inhibiting the migration of microvascular endothelial cells, or function as competitive inhibitors of the binding site between VEGF and VEGF receptor 2, TIMP-1 act as angiogenesis inhibitors (76–78). Recent studies demonstrate significant differences in plasma concentrations of MMP-9, TIMP-1, and TIMP-3 proteins in patients with AMD: higher levels of TIMP-1 and MMP-9 in patients with geographic atrophy (GA) and lower levels of TIMP-3 and lower TIMP-3/MMP-2 ratios in patients with CNV (79). In this particular experimental study, TIMP-1 was reported to be upregulated in the aqueous humor of patients in the poor/non-responsive group, a finding that is still poorly defined in the literature.

Metallothionein 1G (MT1G) is a cysteine-rich, low molecular-weight metalloprotein. In mammals, it plays multiple roles, such as the detoxification of heavy metals, the regulation of essential metal homeostasis, and the protection against oxidative stress. This protein was upregulated in this study's control group, in agreement with the literature, which correlates it with retinal neuroprotection (80).

The phosphorylation of protein kinases is an important mechanism for endothelial cell proliferation and differentiation because the activity of tyrosine kinase receptors is essential for angiogenesis (81). The tyrosine kinase receptor (KDR) and VEGF receptor are the key factors in the pathogenesis of choroidal neovascularization in AMD (82–84). In this study, a type-III tyrosine KDR and vascular endothelial growth factor receptor 1 (VEGFR1) were reported to be upregulated in the poor/non-responsive group and downregulated in the good responsive group.

The ubiquitin–proteosome system is one of the primary components which is responsible for the degradation of proteins that are damaged or unnecessary to cellular metabolism and are produced by heterophagy of the external segments of photoreceptors (85). Ubiquitin carboxy-terminal hydrolase (UCH) was reported to be upregulated in the control group and downregulated in the good responsive group. This finding contradicts those described by Gleen et al. (86).

### Sequential Analysis in Patients With AMD: Resistance to the Gold-Standard Treatment

Excluding the control group, proteins with statistical significance and discriminatory capability as possible biomarkers were identified among the groups of patients with AMD. Two proteins, –LCN1 and the heavy chain of IGHM, were upregulated in the good responsive group and downregulated in the anti-VEGF poor/non-responsive group. On the other hand, for three other proteins, plasma kallikrein (KLKB1), kinase insert domain receptor (KDR), and vascular endothelial growth factor receptor 1 (VEGFR-1), the opposite has been observed.

Indeed, LCN1 and the heavy chain of IGHM were upregulated in the treatment-naive group and downregulated in the poor/non-responsive group. Specifically due to LCN1, its correlation with AMD is consistently established in the literature. According to Ghosh et al. (87), LCN1 acts as a marker of early AMD, particularly when lysosome-mediated clearance is compromised in the RPE, which contributes to the induction of a chronic inflammatory response. This hypothesis had been demonstrated in an animal AMD model (88). There are few studies on the recent literature regarding the relationship between the heavy chain of IGHM and ophthalmological diseases. However, researchers have already demonstrated the association between this protein and rare presentations of hematological diseases (benign monoclonal gammopathy and agammaglobulinemia 1) (89, 90).

For the three proteins that were upregulated in the poor/non-responsive group and downregulated in the good responsive group, the literature shows that one of them (plasma kallikrein, KLKB1) is directly linked to inflammatory processes and two of them (kinase insert domain receptor, KDR and vascular endothelial growth factor receptor 1, VEGFR-1) are linked to vascular and endothelial growth events. By characterizing a gradual loss of drug efficacy (anti-VEGF) over time, during the gold-standard therapy for neovascular AMD, some researchers introduced a new concept of treatment-resistance in the context of AMD. Tolerance to medication is characterized by a slow loss of efficacy over time (91). The effect of the drug can be improved if the dosage is increased or if the drug is administered at shorter intervals. On the other hand, the efficacy is not restored if anti-VEGF treatment is stopped temporarily (91). In this research, this difference in the number of receptors in poor/non-responsive patients not only might explain the difficulty in controlling the subretinal neovascular membrane regime, but also may corroborate the explanation regarding the pathological tolerance that some patients develop to the gold-standard treatment of AMD. Since VEGFR-1 is upregulated in patients of the poor/non-responsive group, this feature may explain, at least in part, the persistence of disease activity, even with the usage of anti-VEGF in patients with AMD.

Among the 39 proteins selected in this study, the most promising were those with upregulated and higher relative intensity (VIP score >1), feasible access to serum matrix, and relevant metabolic importance in choroidal neovascularization impaired pathways.

By their importance in the visual function, the crystalline α-, β-, and γ- (such as the α-crystalline, CRYAB; β-crystalline 1, CRBB1; and γ-crystalline C, CRYGC) stand out. In the lipid metabolism pathway, important in early diagnosis, apolipoprotein A-I isoform CRA_A (APOA1), which is correlated with the formation of drusen, appears with high discriminant, along with phosphoinositide phospholipase C (PLC), which features the greatest fold change, and retinol-binding protein 3 (RBP3), for its potential in serum documentation by self-antibodies.

In the oxidative stress pathway, CAT is the protein with the highest relative intensity and the highest discriminatory capability within the phenotypic conditions (VIP score). In the complement activation pathway, this study corroborates with the descriptions in the literature, with the groups of patients with AMD showing, in general, a relative increase compared with the control group. All proteins described in this study and involved in this specific pathway may be potential biomarker candidates.

In the inflammatory pathway, enolase 1 (HEL-S-17) is noteworthy, because it is presented within the 30 proteins with the highest VIP score. Finally, in the angiogenic pathway, VEGFR 1 is highlighted as an important effector, and plays a core role in the choroidal neovascularization pathophysiology. Although, there are discrepancies with the literature data, tissue inhibitor of metalloproteins 1 (TIMP 1) is displayed at the VIP score chart. Nonetheless, further studies are the requirement to a deeper understanding of this effector.

Although under treatment, an important percentage of the population with nAMD remains with the disease inactivity. This aspect requires a deeper understanding of alternative pathways for choroidal neoangiogenesis. Nowadays, high-performance analytical experiments pursue diagnostic and therapeutic means through additional pathways in the pathophysiogenesis of nAMD. In this study, the proteins selected may contribute as potential biomarkers for early diagnosis and even new adjuvant therapeutic targets.

A more expanded research analysis should be performed as well, and validation studies in large cohorts are still required to assess the diagnostic and prognostic capability of the protein classes selected herein.

## Data Availability Statement

The datasets for this study can be found in the Repository MassIVE (https://massive.ucsd.edu/ProteoSAFe/dataset.jsp?task=852e34f8818041e3a434132163460c89).

## Ethics Statement

The studies involving human participants were reviewed and approved by Ethics Committee: Plataforma Brasil/Hospital Oftalmológico De Brasília (under the number CAAE 64921317.1.0000.5667). The patients provided their written informed consent to participate in this study.

## Author Contributions

BC has contributed to conceptualizing, drafting, writing, experiment analysis, funding, and final revision of the manuscript. FC has organized the data and tables. RO has involved in experiment and data analysis, organizing figures and reference revision. ON and CR have contributed on funding and final revision. WF has performed experiment and data analysis. MS has contributed on funding and infrastructure. MÁ has contributed to conceptualizing, infrastructure, mentoring, and final revision. AM has contributed to conceptualizing, drafting, experiment and data analysis, writing, mentoring, final revision, and supervision of the manuscript. All authors contributed to the article and approved the submitted version.

## Conflict of Interest

The authors declare that the research was conducted in the absence of any commercial or financial relationships that could be construed as a potential conflict of interest.

## Publisher's Note

All claims expressed in this article are solely those of the authors and do not necessarily represent those of their affiliated organizations, or those of the publisher, the editors and the reviewers. Any product that may be evaluated in this article, or claim that may be made by its manufacturer, is not guaranteed or endorsed by the publisher.
