# Supplementary material for: Novel Possible Protein Targets in Neovascular Age-Related Macular Degeneration: A Pilot Study Experiment
Source: Front Med (Lausanne). 2022 Jan 27;8:692272. doi: 10.3389/fmed.2021.692272 (PMC8828634; doi:10.3389/fmed.2021.692272)
Supplement: Supplementary file 1 [file Table_1.DOCX]

Supplementary Material

# Supplementary Figures and Tables

## SupplementaryTables

**Supplementary Table1.** Response to anti-VEGF therapy in nAMD adapted by Amoaku et al. (20).

| Response | Morphology | Functional |
| --- | --- | --- |
| Good | Absence of SRF, IRF, IRC or a reduction of CRT >73% of the baseline values. | Improvement in VA >5 letters from the baseline (ceiling effect in eyes with good starting VA defined as ETDRS 70 letters or above). Pay more attention to morphological features if VA is good esp>70). |
| Partial | Reduction of CRT of between 25 and 75% of the baseline values, and/or persistence of SRF, IRF, IRC and or appearance of new IRC, IRF and SRF. | Change in VA of 1-5 letters from the baseline. |
| Poor | Between 0 and 25% reduction in CRT and/or persistence of SRT, IRF, IRC and/or appearance of new IRC, IRF and SRF. | Change in VA of 0-4 letters from the baseline. |
| Non-response | Unchanging or increasing CRT, SRF, IRF and/or PED | Change > - 5 letters is decline in VA from the baseline from 1 month after third initiation injection. |

CRT, central retinal thickness in the central 1000-µm subfield; IRC, intraretinal cysts; IRF, intraretinal fluid; SRF, subretinal fluid; VA, visual acuity. Notes: 1. Retinal atrophy/ thinning and/ or subretinal fibrosis do not imply poor response, but confound VA. Similarly, minimal change or fluid over scar tissue etc. may not imply poor response. These may result from longstanding disease, rather than treatment outcomes. 2. Outer retinal tubulations (ORT) do not represent active fluid leakage. 3. PED presence – evidence to date does not indicate that flattening of PED determines outcomes, however, PED progression indicates active disease and requires ICGA to exclude IPCV, and/or consideration of treatment change. 4. Morphological and functional features (responses) may not correlate. 5. Primary response determined after initiation phase, ie. at first visit after the 3rd initiation injection. 6. Secondary response determined any time from 1 month after the 3rd initiation injection (months 4-11). 7. Late response determined at month 12 or after.
